# Influence of Peanut, Sorghum, and Soil Salinity on Microbial Community Composition in Interspecific Interaction Zone

**DOI:** 10.3389/fmicb.2021.678250

**Published:** 2021-05-24

**Authors:** Xiaolong Shi, Xinhua Zhao, Jinyao Ren, Jiale Dong, He Zhang, Qiqi Dong, Chunji Jiang, Chao Zhong, Yufei Zhou, Haiqiu Yu

**Affiliations:** College of Agronomy, Shenyang Agricultural University, Shenyang, China

**Keywords:** bacteria, fungi, mixed cultivation, phylum, rhizosphere, soil salinity

## Abstract

Soil microorganisms play important roles in crop production and sustainable agricultural management. However, soil conditions and crop selection are key determining factors for soil microbial communities. This study investigated the effect of plant types and soil salinity on the microbial community of interspecific interaction zone (II) based on the sorghum/peanut intercropping system. Microbial community diversity and composition were determined through PacBio single molecule, real-time sequencing of 16S rDNA and internal transcribed spacer (ITS) genes. Results showed Proteobacteria, Bacteroidota, and Acidobacteriota to be the dominant bacterial phyla in IP, II, and IS, whereas Ascomycota, Basidiomycota, and Mucoromycota were the dominant fungal phyla. Under salt-treated soil conditions, the plants-specific response altered the composition of the microbial community (diversity and abundance). Additionally, the interspecific interactions were also helpful for maintaining the stability and ecological functions of microbial communities by restructuring the otherwise stable core microbiome. The phylogenetic structure of the bacterial community was greatly similar between IP and II while that of the fungal community was greatly similar between IP and IS; however, the phylogenetic distance between IP and IS increased remarkably upon salinity stress. Overall, salinity was a dominant factor shaping the microbial community structure, although plants could also shape the rhizosphere microenvironment by host specificity when subjected to environmental stresses. In particular, peanut still exerted a greater influence on the microbial community of the interaction zone than sorghum.

## Introduction

Soil salinization is a compelling environmental challenge that limits the growth and productivity of crops worldwide, since 20% of the world’s arable land and 33% of the irrigated land are affected by soil salinization (Kashyap et al., 2020). Development and utilization of salinized soils is an important strategy to increase the cultivated land area, which is of great significance to meet the food demands of an increasing global population (Amelung et al., 2020). However, long-term continuous cropping can result in reduction of crop yield, accumulation of soil-borne plant pathogens (Klein et al., 2016; Cui F. et al., 2018), and disruption of soil microbial community (Liu et al., 2019; Tang et al., 2020). Particularly, in salinized soils, monoculture farming faces a double combination of excess salinity and continuous cropping obstacles. To overcome these problems, intercropping can be considered a novel approach to increase the efficiency of resource utilization, management of soil-borne diseases, and tolerance of farmland ecosystem stress (Li Q. et al., 2016; Guo et al., 2018; Dang et al., 2020).

In the intercropping system, roots of different types of plants are in direct contact with the soil, and they directly or indirectly affect the development of soil microbiota in the rhizosphere and in bulk soil by means of root exudates (Haichar et al., 2014). In addition, interspecific root interaction can create a relatively stable microhabitat through resource competition and mutual promotions (Aguilera et al., 2017; Cavalieri et al., 2020). The beneficial soil eco-quality and ecosystem services in the intercropping model are mainly driven by soil microbial activities (Granzow et al., 2017; Nivelle et al., 2018). Naturally, the adaptive evolution and composition of microbial communities are governed by plant species, developmental stages, and environmental factors (Ma et al., 2016). Rhizosphere microorganisms, especially beneficial bacteria and fungi, co-evolve with their host to adjust the community structure based on the specific environments, ultimately improving plant performance and enhancing plant resistance to stress conditions both directly and indirectly (Grover et al., 2011; Zhao et al., 2021). For example, Li X. et al. (2016) found that mulberry-soybean intercropping increases the abundance of beneficial bacteria (phosphate-solubilizing bacteria) in intercropping samples under salt-alkali soil condition. Zeng et al. (2019) indicated that the rhizosphere interactions in a mixture model are helpful in recruiting beneficial microbes, which are closely related to the improvement of plant quality in contaminated soil. Additionally, other typical systems of crop production, such as maize-peanut intercropping (Li et al., 2018), maize-soybean intercropping (Chang et al., 2020), and millet-sorghum intercropping (Iijima et al., 2016), have provided evidence of change of microbial communities and enhancement of biotic or abiotic stress tolerance. Thus, the recruitment of specific microbial communities under particular production conditions is an interesting topic.

Intercropping of legumes and cereals is one of the most common interspecies combinations in agricultural production, with their niche complementarity and interspecific positive interactions enabling better adaptation to environmental changes and stresses (Brooker et al., 2015). Sorghum (*Sorghum bicolour* L.) possesses stronger salt tolerance (Huang, 2018), while peanut (*Arachis hypogaea* L.) is also considered to be a moderately salt-tolerant crop (Cui J. et al., 2018). They can adapt to arid and barren soil conditions, and are widely cultivated in the marginal lands in northeast China (Guo et al., 2021); therefore, they are regarded as a pioneer crop that can be grown in saline soils. However, the influence of micro-ecological soil environment on the sorghum and peanut intercropping system has not been fully investigated yet, especially under salinity stress conditions.

The present study aimed to illustrate the variation of bacterial and fungal communities in peanut-sorghum intercropping systems by genetic amplification and sequencing of the 16S rDNA and internal transcribed spacer (ITS) regions. Further, it aimed to investigate the recruitment of specific microbial communities by various plant species and understand the deterministic role of salinity in shaping the microbial communities. We hypothesized that (1) the peanut and sorghum plants might differentially affect the soil microbial community in the interspecific interaction zone (II) and (2) microbial community could undergo species-specific changes under salt-treaded soil conditions. This study could provide insights into the optimization of cropping systems and improvement of farmland ecological stability of in salinized soils.

## Materials and Methods

### Experimental Design

Experiments were conducted in the experimental field station at Shenyang Agricultural University, Shenyang, China for two consecutive years during the growth seasons of 2018 and 2019. Sorghum (Liaoza 15) and peanut (Huayu 25) plants were grown in large wooden planting boxes 1.00 m × 1.00 m × 0.60 m in dimension. Experimental soil was collected from the 0 to 20 cm thick cultivation layer of the experimental field and classified brown soil with 96.70 mg hydrolyzable N kg^–1^, 27.50 mg available P kg^–1^, 117.9 mg available K kg^–1^, 15.17 g organic matter kg^–1^, 680 mg soluble salinity kg^–1^, and pH 6.5. Before sowing, the soil salinity concentration was adjusted to 2.5 g (NaCl) kg^–1^ to mimic a moderate soil salinity level in the field (S, 0.25% NaCl) (Shi et al., 2020), and untreated soil served as the control (N, 0.00% NaCl). The soil (approximately 800 kg) was then put into the wooden planting boxes, and the same doses of 75 kg N ha^–1^, 120 kg P_2_O_5_ ha^–1^, and 170 kg K_2_O ha^–1^ were applied as the basal fertilizer to each box. After incubating the soil in the natural environment for about 30 days upon NaCl addition, and when the soil moisture was ideal and appropriate, five sorghum plants and six peanut plants, in each box, were intercropped under two different soil conditions. The row spacing was 40 cm and each treatment was conducted in triplicate. The same field management was implemented for all treatments during the experimental period.

### Soil Sampling

After 2 years of continuous planting, soil samples were collected from the peanut rhizosphere (IP), sorghum rhizosphere (IS), and II at 60 days after germination in 2019 (Supplementary Figure 1). Soil samples were collected using a sterilization shovel from the depths of 0–20 cm (after removal of 5 cm of the soil surface) of the rhizosphere and interaction zone. Loosely adhered soil samples from the root surface were removed by gentle shaking, and then the tightly bound rhizosphere soil was removed using a sterilized brush (changed between samples). Samples of rhizosphere soil were collected and thoroughly mixed in order to obtain a composite sample. The composite samples, representing each site, were passed through a 2 mm sieve, collected in 15 mL sterile centrifuge tubes on ice, transferred to the laboratory within 8 h, and kept frozen at −80°C until DNA extraction. The remaining soil sample was air dried for its physiochemical analyses.

### DNA Extraction and High-Throughput Sequencing

Total microbial community DNA was isolated from 0.25 g of soil per sample using the PowerSoil^®^ DNA Isolation kit (*MO BIO Laboratories* Inc., Carlsbad, CA, United States) according to the manufacturer’s instructions. Quality and concentration of the extracted DNA were checked by 0.8% agarose gel electrophoresis and ultraviolet spectrophotometry, respectively. High-quality DNA samples were amplified using primers targeting the full-length regions of the bacterial 16S rDNA gene and fungal ITS sequences. For bacterial community, the bacterial 16S rDNA genes were amplified using the universal bacterial primers 27F (5′-AGRGTTYGATYMTGGCTCAG-3′) and 1492R (5′-RGYTACCTTGTTACGACTT-3′). For the fungal community, ITS sequences were amplified using the primers ITS1F (5′-CTTGGTCATTTAGAGGAAGTAA-3′) and ITS4R (5′-TCCTCCGCTTATTGATATGC-3′). PCR amplification was performed in a total reaction volume of 20 μL, which contained 10 μL buffer, 0.4 μL DNA polymerase, 4 μL dNTP, 1 μL of each primer (10 μM), and 50 ng genomic DNA. The PCR was set up as follows: an initial denaturation step for 5 min at 95°C, followed by 30 cycles of denaturation at 95°C for 30 s, annealing at 50°C for 30 s, and extension at 72°C for 1 min, and a final extension step at 72°C for 7 min. After electrophoresis of the PCR products, they were purified, quantified, and homogenized to create a SMRTbell sequence library, and the marker gene was sequenced using the PacBio single molecule, real-time (SMRT) sequencing technology (Pacific Biosciences, Menlo Park, CA, United States). All the above-mentioned procedures were completed by Biomarker Technologies Corporation (Beijing, China).

### Data Pre-processing and Sequencing Data Bioinformatics Analysis

Circular consensus sequencing (CCS) reads were obtained with the SMRT Link v8.0 after correcting the raw sub-reads. CCS reads were barcode-identified and length-filtered; the chimeras were removed thereafter using UCHIME v.8.1 (Edgar et al., 2011), and finally optimized-CCS reads were obtained. Optimized-CCS reads were clustered into operational taxonomic units (OTUs) at 97% similarity using the USEARCH v.10.0 software (Edgar, 2013), and the representative OTU sequences were annotated using the SILVA bacterial 16S rRNA database (Release132)^1^ (Quast et al., 2013) and the UNITE fungal ITS database (Release 8.1)^2^ (Kõljalg et al., 2013) by a QIIME-based wrapper of RDP-classifier v.2.2 with a confidence cutoff of 0.8. The detected communities were identified and annotated at different taxonomic levels (phylum, class, order, family, genus, and species). Further analysis was done to calculate alpha diversity and richness of OTUs, and the community composition of each sample was determined at different classification levels. Finally, based on the above analysis, alpha diversity index was applied to analyze the microbial diversity, including the Chao1, Shannon, and Ace indices and rarefaction curves, which were obtained with Mothur software (version 1.30.1)^3^. The principal co-ordinates analysis (PCoA) was applied to reduce the dimension of the original variables based on the Binary-Jaccard distances using R v.3.5.3. The Circos graph was built using Circos-0.67-7 software^4^. Linear discriminant analysis (LDA) coupled with effect size measurements (LEfSe) analysis was applied to search for statistically different biomarkers between different groups using LEfSe software^5^. The relationship between the soil microbial community structures and soil environmental factors was analyzed by redundancy analysis (RDA) using Canoco software v.4.5.

### Statistical Analysis

Differences across the treatments were calculated and analyzed statistically using one-way analysis of variance (ANOVA) and the least significant difference (LSD) multiple range test (*p* < 0.05). The statistical analyses were conducted using GraphPad Prism version 5.1 (GraphPad Software, San Diego, CA, United States) and the SPSS software version 19.0 (SPSS, Inc., Chicago, IL, United States).

## Results

### General Characteristics of PacBio SMRT Sequencing Based 16S rDNA and ITS Datasets

Each sample was identified and distinguished according to the barcode-labeled sequence; 376,040 CCS reads with bacterial species annotations were obtained from 18 soil samples, with an average of 20,891 CCS reads per sample (range: from 19,854 to 21,200). For fungi, 144,040 CCS reads were obtained, with an average of 8,002 CCS reads per sample (range from 7,911 to 8,109). After barcoded-CCS read quality filtering and removal of chimeras, an average of 19,904 bacterial optimized-CCS reads were generated per sample, with 95% effective sequences. An average of 7,844 fungal optimized-CCS reads per sample were obtained with 98% effective sequences (Supplementary Table 1).

### Venn Diagrams Analysis of OTUs

**FIGURE 1:**
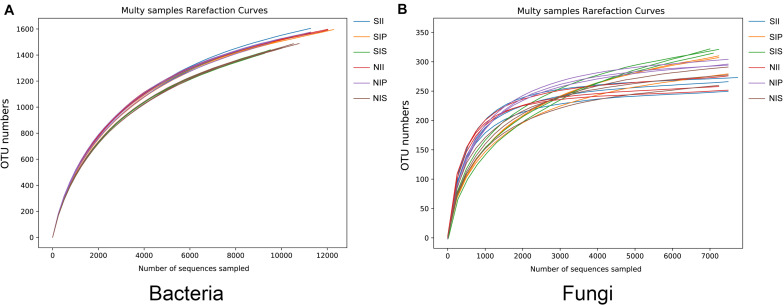
Rarefaction curves of the peanut rhizosphere (IP), sorghum rhizosphere (IS), and interspecific interaction zone (II) microbial communities under different soil conditions (S, 0.25% salt-treated soil condition; N, normal soil condition). **(A)** Rarefaction curves of bacterial community. **(B)** Rarefaction curves of fungal community.

**FIGURE 2:**
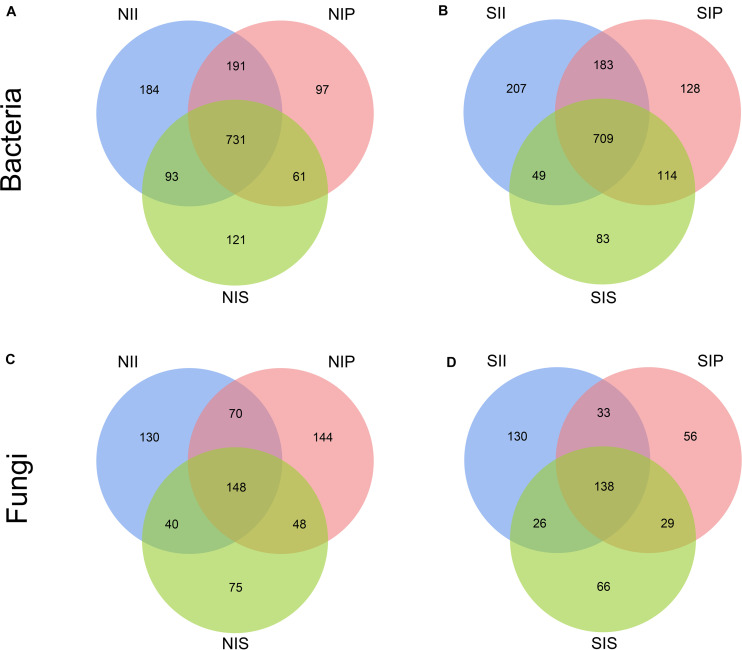
Venn diagrams showing the unique and shared operational taxonomic units (OTUs) (5% distance level) between the peanut rhizosphere (IP), sorghum rhizosphere (IS), and interspecific interaction zone (II) (S, 0.25% salt-treated soil condition; N, normal soil condition). **(A)** Venn diagrams of bacterial operational taxonomic units (OTUs) under normal soil condition. **(B)** Venn diagrams of bacterial OTUs under salt-treated soil condition. **(C)** Venn diagrams of fungal OTUs under normal soil condition. **(D)** Venn diagrams of fungal OTUs under salt-treated soil condition.

Rarefaction curve analysis showed that when the sample sequencing quantity of bacteria reached 4,000 and that of fungi reached 1,000, the curve gradually flattened (Figure 1). On an average, 1,531 bacterial OTUs and 284 fungal OTUs were obtained from the 18 samples with an OTUs coverage of over 95 and 98%, respectively, indicating that the sequencing depth of all the samples was reasonable and the sequencing date reflected information about the microbial community of the sample. The Venn diagrams depicted the number of OTUs unique to each sample or shared among multiple samples and intuitively displayed an OTU overlap across samples. Bacterial OTU analysis indicated that 731 OTUs were common to all samples; the number of bacterial OTUs shared by NIP and NII was 191 (Figure 2A). Fungal OTU analysis indicated that 148 OTUs were common to all samples; the number of fungal OTUs shared by NIP and NII was 70 (Figure 2C). In contrast, 93 bacterial OTUs and 40 fungal OTUs were shared by NIS and NII, respectively. Under salt-treated soil condition, the number of OTUs common to all samples was reduced, and the number of bacterial and fungal OTUs shared by SIP and SII were still higher those by SIS and SII (Figures 2B,D).

### Richness and Diversity Analyses of Bacterial and Fungal Communities

For the bacterial community, Ace and Chao1 indices in IP and II were higher than those in IS. Under salt-treated soil conditions, the Ace and Chao1 indices increased in SIP and SII, but decreased in SIS. The fungal Ace and Chao1 indices of each sample followed the order NIP > NIS > NII. Under salt-treated soil conditions, Ace and Chao1 indices decreased in SIP and SII, but increased in SIS (SIS > SIP > SII). In general, the Shannon index of the bacterial and fungal communities in IP and II were significantly higher than that in IS. The Shannon index of the microbial communities reduced under salt-treated soil conditions, and the reductions of Shannon index of the fungal community in SIP and SII were the most prominent (Table 1).

**TABLE 1:** Diversity and richness of the peanut rhizosphere (IP), sorghum rhizosphere (IS), and interspecific interaction zone (II) soil microbial communities at the phylum and genus levels under different soil conditions (S, 0.25% salt-treated soil condition; N, normal soil condition).

**Microorganism**	**Sample**	**97%**				
		**OTU**	**Ace**	**Chao1**	**Shannon**	**Coverage**
**Bacteria**	SII	1577	1806.3855ba	1770.6447^b^	6.5279^a^	0.9685
	SIP	1565	1822.2369^a^	1820.3562^a^	6.5232^a^	0.9663
	SIS	1446	1754.6625^c^	1738.1221^b^	6.3103^b^	0.9590
	NII	1577	1778.896bca	1763.4066^b^	6.5686^a^	0.9711
	NIP	1538	1786.7962bca	1757.3794^b^	6.5433^a^	0.9645
	NIS	1483	1773.6078cb	1750.8530^b^	6.3187^b^	0.9634
**Fungi**	SII	221	224.4201^d^	227.0040^d^	4.0638cb	0.9987
	SIP	297	321.498cb	317.3411cb	3.9223^c^	0.9942
	SIS	345	400.2841^a^	394.6947^a^	4.1535cb	0.9897
	NII	238	241.4811^d^	244.6667^d^	4.6443^a^	0.9985
	NIP	326	333.4707^b^	336.3298^b^	4.7365^a^	0.9974
	NIS	278	286.8004^c^	286.9774^c^	4.2715^b^	0.9970

*Different lowercase letters in the same column indicate significant differences between the groups at a significance level of p < 0.05. OTU, operational taxonomic unit.*

### Equalization Analysis Between Bacterial and Fungal Community Structures

**FIGURE 3:**
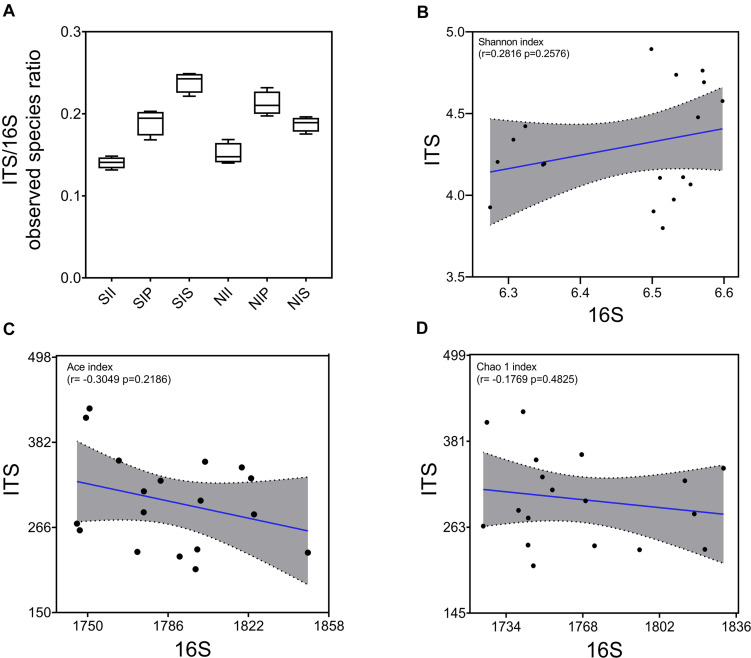
Equalization analysis of the peanut rhizosphere (IP), sorghum rhizosphere (IS), and interspecific interaction zone (II) microbial communities under different soil conditions (S, 0.25% salt-treated soil condition; N, normal soil condition). **(A)** Ratio of bacterial and fungal operational taxonomic units (OTUs). **(B)** the correlation between bacterial and fungal Shannon indices. **(C)** the correlation between bacterial and fungal Ace indices. **(D)** the correlation between bacterial and fungal Chao1 indices.

Under salt-treated soil conditions, the ITS/16S ratio decreased in SIP and increased significantly in SIS, with no obvious change in SII (Figure 3A). The correlation between fungal and bacterial diversities was examined by Pearson’s correlation analysis, and a lower correlation between the Shannon diversity indices of the bacterial and fungal communities was found (*r* = 0.2816, *p* = 0.2576) (Figure 3B). However, the Ace and Chao1 indices of the bacterial community were negatively correlated with those of the fungal community (*r* = −0.3049 and −0.1769, respectively) (Figures 3C,D). This showed that the overall equalization of the bacterial and fungal communities was affected by both plant species and soil salinity, thereby indicative of the major contributor to the change in the microbial equilibrium. Variation in richness of microbial species was the principal cause for the change in the microbial equilibrium.

### Analysis of the Bacterial and Fungal Community Composition

**FIGURE 4:**
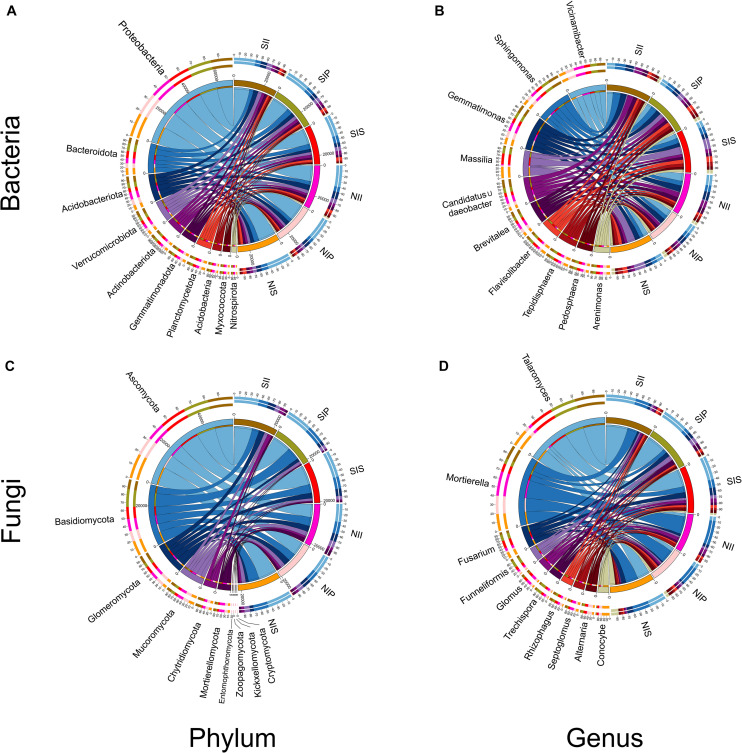
Relative abundance of the peanut rhizosphere (IP), sorghum rhizosphere (IS), and interspecific interaction zone (II) microbial communities at the phylum and genus levels under different soil conditions (S, 0.25% salt-treated soil condition; N, normal soil condition). **(A)** Bacterial community composition at the phylum level. **(B)** Bacterial community composition at the genus level. **(C)** Fungal community composition at the phylum level. **(D)** Fungal community composition at the genus level.

From the phylum to genus level, the different soil conditions and plant types have different influences on the bacterial and fungal community structures. At the phylum-level, Proteobacteria, Bacteroidota, Acidobacteriota, and Verrucomicrobiota were the dominant bacterial communities in each sample, with the relative abundance of Proteobacteria being the highest (36.4%) (Figure 4A). Furthermore, the relative abundances of Acidobacteriota, Gemmatimonadota, and Myxococota significantly decreased in salt-treated soil, whereas those of Bacteroidota, Verrucomicrobiota, and Actinobacteriota were significantly increased. At the bacterial genus level, the relative abundances of *Sphingomonas*, *Massilia*, *Candidatus_Udaeobacte*r, *Brevitalea*, *Flavisolibacter*, *Tepidisphaera*, and *Pedosphaera* increased due to salt treatment while those of *Vicinamibacter* and *Gemmatimonas* decreased (Figure 4B). Among these, *Sphingomonas*, *Candidatus_Udaeobacter*, and *Brevitalea* were detected with the highest abundances in II, and *Massilia*, *Flavisolibacter*, *Tepidisphaera*, and *Arenimonas* were the most abundant in IS (Supplementary Table 2). Concerning the fungal community, Ascomycota, Basidiomycota, Glomeromycota, and Mucoromycota were the most abundant phyla. Of these, the abundances of Ascomycota was significantly higher levels in IS than in IP and II (Figure 4C). Compared with the normal soil conditions, Mucoromycota, Mortierellomycota, and Kickxellomycota abundances decreased after salt-treatment, but the abundance of Ascomycota increased in each sample, with the most significant increase (approximately 35.7%) being in SIP and NII. Under normal soil conditions, a low abundance of Entomophthoromycota and Kickxellomycota was detected in NIP and NII, and NIP, respectively, while none was detected in the salt-treated soil (Supplementary Table 3). Fungal genus *Talaromyces* had higher abundance in NIS than in NII and NIP, and the abundance of *Talaromyces* increased 602.4, 435.5, and 45.3% in SII, SIP, and SIS, respectively (Supplementary Table 3). The abundance of *Mortierella* and *Conocybe* decreased in SII, SIP, and SIS, with the reduction in the former being the most prominent in SIP and that in the latter being the most prominent in SIS. In addition, the abundances of *Fusarium*, *Funneliformis*, and *Trechispora* were significantly increased in SIP and SIS, although they were insignificant in SII (Figure 4D).

### Beta Diversity Analysis of Bacterial and Fungal Communities

**FIGURE 5:**
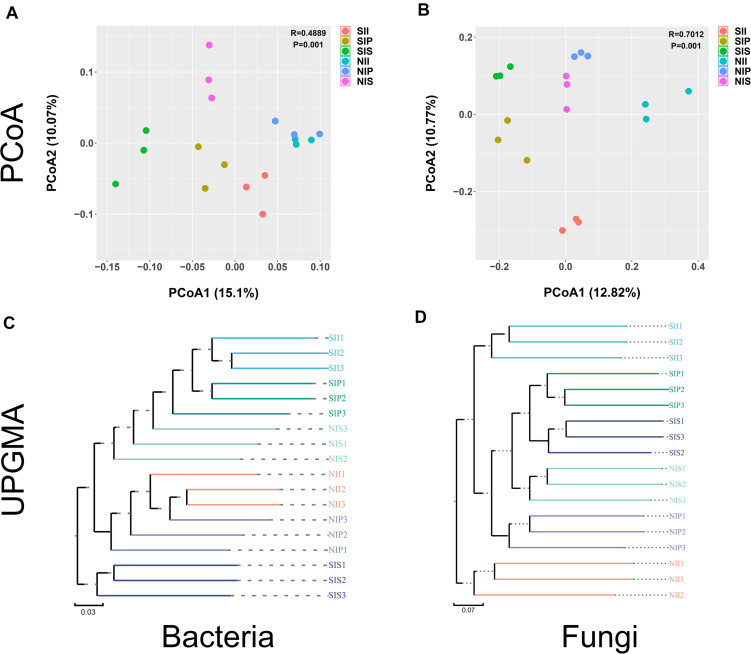
Principal coordinate analysis and unweighted pair group method with arithmetic mean analysis of the peanut rhizosphere (IP), sorghum rhizosphere (IS), and interspecific interaction zone (II) microbial communities at the phylum level under different soil conditions (S, 0.25% salt-treated soil condition; N, normal soil condition). **(A)** PCoA of bacterial community composition. **(B)** PCoA of fungal community composition. **(C)** UPGMA cluster analysis of bacterial community composition. **(D)** UPGMA cluster analysis of fungal community composition.

Both unweighted pair group method with arithmetic mean (UPGMA) analysis and PCoA analyses revealed distinct differences in the bacterial and fungal community structures across the different samples. For bacterial community, large differences in the microbial community structures were observed between the salt-treated soil and normal soils. Meanwhile, PC1 and PC2 components of PCoA together explained 25.15% of the variance. The bacterial community structures from NII and NIP clustered together and were completely separated from that of NIS; however, the phylogenetic distance between SII, SIP, and SIS was relatively large. In comparison, the community structures were more similar between SIP and SII (Figures 5A,C). For fungi, the samples of NII, NIP, and NIS with similar evolutionary processes tended to cluster into the same group, which suggested that they had a similar microbial community. However, the difference in the fungal community between SII, SIP, and SIS was enhanced remarkably under salt-treated soil conditions, and the PC1 and PC2 components together explained 23.59% of the variance (Figure 5B). UPGMA dendrograms showed that the salt-treated soil samples and normal soil samples were grouped into two major clusters, and the fungal community composition had a relatively higher similarities between IP and IS under different soil conditions (Figure 5D).

### Redundancy Analysis of Microbial Community and Soil Properties

**FIGURE 6:**
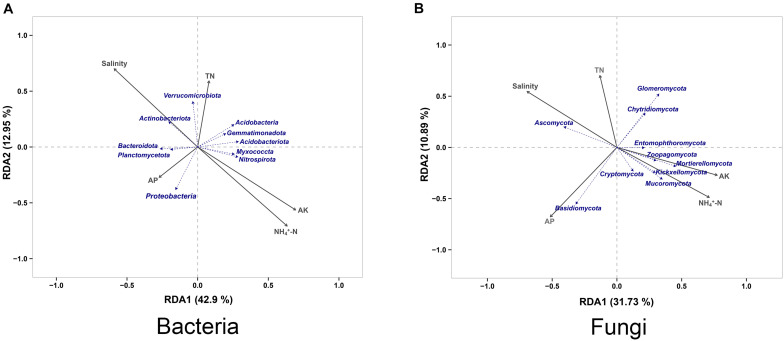
Redundancy analysis of the peanut rhizosphere (IP), sorghum rhizosphere (IS), and interspecific interaction zone (II) microbial communities at the phylum level under different soil conditions (S, 0.25% salt-treated soil condition; N, normal soil condition). TN, total nitrogen; NH_4_^+^-N, ammonium nitrogen; AP, available phosphorus; AK, available potassium. **(A)** Redundancy analysis (RDA) of bacterial community. **(B)** Redundancy analysis (RDA) of fungal community.

Redundancy Analysis (RDA) was applied to reveal the relationships between soil properties and the microbial community, and we found that salinity, available potassium (AK) and ammonium nitrogen (NH_4_^+^-N) contents to played a key role in structuring the microbial communities. For bacterial community, Actinobacteriota had a higher correlation with salinity compared with other species, whereas Verrucomicrobiota was more affected by the total nitrogen (TN) content compared with other species. Bacteroidota and Planctomycetota had similar environmental attribute values, which were affected by the available phosphorus (AP) content like Proteobacteria. In addition, Myxococcota and Nitrospirota had similar environmental attribute values, the AK and NH_4_^+^-N contents showing a considerable level of impact on them (Figure 6A). For fungal community, Ascomycota showed a high correlation with the salinity. Chytridiomycota and Glomeromycota were mainly affected by the TN content of the soil, while Basidiomycota was mainly related to the AP content. The AK and NH_4_^+^-N contents had a greater impact on fungal community species, of which Mucoromycota, Kickxellomycota, Mortierellomycota, and Zoopagomycota were the most severely affected (Figure 6B).

### Differential Abundance of Microbial Taxa Across the Groups

**FIGURE 7:**
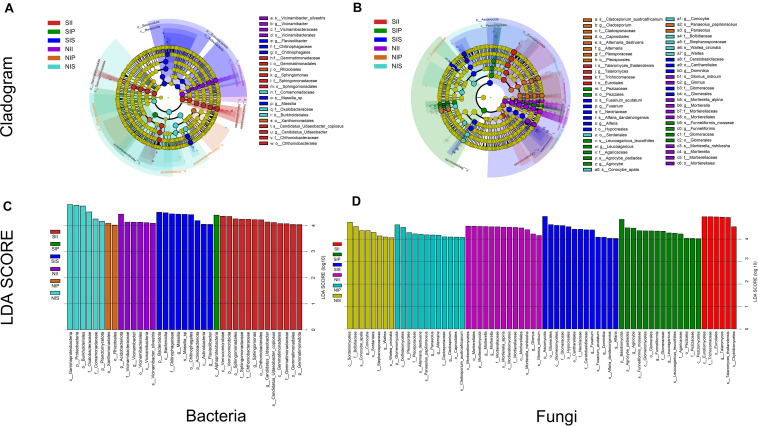
Linear discriminant analysis effect size of the peanut rhizosphere (IP), sorghum rhizosphere (IS), and interspecific interaction zone (II) microbial biomarkers under different soil conditions (S, 0.25% salt-treated soil condition; N, normal soil condition). **(A)** Bacterial core community composition. **(B)** Fungal core community composition. **(C)** LDA scores of biomarker bacteria. **(D)** LDA scores of biomarker fungi.

From phylum level to species level, different plant species had specific effects on both the bacterial and fungal communities under different soil conditions. For bacteria, three microbial taxa were significantly enriched in each sample, namely Proteobacteria, Planctomycetota, and Acidobacteriota under normal soil conditions. In NIP, the dominant class was Alphaproteobacteria, whereas in NIS, it was Betaproteobacteria. Moreover, Acidobacteriota (from phylum to species) was abundant in NII. Under salt-treated soil conditions, core taxa of SIP did not vary significantly with soil conditions, whereas that of SIS and SII showed distinct shifts, Bacteroidetes (from phylum to genus) was prominent in SIS, and Verrucomicrobiota (from phylum to species) and Gemmatimonadota (from phylum to family) were significantly enriched in SII (Figures 7A,C). For fungi, the dominant phyla in the fungal community exhibited no detectable differences between NIP and NIS, although significant taxonomic differences were present. Ascomycota (e.g., Dothideomycetes and Capnodiales) and Basidiomycota (*Panaeolus*) were prominent in NIP, whereas Ascomycota (e.g., Sordariomycetes) and Basidiomycota (e.g., *Waitea* and *Conocybe*) were enriched in NIS. The core taxa in NII showed substantial differences compared with other samples, and multiple taxa within the phylum Mucoromycota to be enriched in NII. Under salt-treated soil conditions, multiple taxa within the phylum Mucoromycota were the most prominent in SIP and SIS. In addition, a large number of taxa within the phylum Ascomycota were recruited by SII, and similar core taxa were also detected between SIP and SIS, although there was a significant taxonomic difference (Figures 7B,D).

## Discussion

Plant-associated microorganisms have recently been studied intensively to determine the influences of the host plant species and their genotypes (Edwards et al., 2018; Tkacz et al., 2020). In this study, the microbial diversity analysis showed that the species richness (Ace and Chao1 indices) and diversity (Shannon index) of microbial communities in IP were significantly higher than that of in IS, especially the fungal community (Table 1). Pertaining to the fungal community, IP had a significantly higher abundance of many core taxa than in IS, including Glomeromycota, Entomophthoromycota, *Funneliformis*, and *Rhizophagus* (Figures 4C,D). However, the abundance of the core bacterial taxa (Bacteroidota, Planctomycetota, and *Massilia*) was higher in IS than in IP (Figures 4A,B). This finding further verified that the ratio of ITS/16S in IP was higher than that in IS (Figure 3A); a low microbial diversity in IS may be the result of highly competitive resident microorganisms (Goerges et al., 2008). Further studies on the manipulation of the root-associated microbiome and recruitment of beneficial microorganisms revealed that the most predominant phylum in the IP and IS in this study was Proteobacteria, although the taxa were significantly different between peanut and sorghum. Rhizobiales (Alpha subclass of the Proteobacteria) was the core taxon in IP, and Burkholderiales (Delta subclass of the Proteobacteria) is the core taxon in IS (Figure 7A). The observed results corroborated those of a previous study, which showed population diversities of partners in N−fixing rhizobium-legume symbiosis to be matched (Igolkina et al., 2019). These results indicated that even in the same microorganism library, plants can still actively recruit plant-associated microorganisms from the soil to construct specific microbial communities.

Mixed cultivation can significantly impact the soil microbial communities, and the interaction across host plant species plays a major role in shaping the soil microbial communities by modifying the rhizosphere microenvironment (Wahbi et al., 2016; Cavalieri et al., 2020). Due to the differences in the root system architecture and exudates of different plant species, the impact of shaping microbial communities in II also differs. This study found Acidobacteriota and Mucoromycota to be the core taxa of bacterial and fungal communities in II, respectively, which was completely different from that in IP and IS (Figure 7A). The variation may be due to a change in the soil microenvironment by interspecific interactions, which determines the differential selection of microbial partners from the soil environment (Dai et al., 2019). Nevertheless, we found a strong species-specific difference in the microbial composition at the OTU level. The Venn diagrams indicated that the number of shared OTUs was significantly higher between IP and II than between IS and II (Figures 2A,C). From the phylogenetics perspectives, the bacterial community composition of IP was similar to that of II (Figure 5C). Moreover, PCoA and UPGMA analysis revealed similarities in the fungal community and phylogenetic processes between IP and IS, which were significantly different from that of the II (Figures 5B,D). Taken together, these findings that peanut exerted a greater influence on the soil bacterial community of II, while the fungal communities were mainly driven by soil properties, and the changes in soil properties are closely related to the species coverage (Fox et al., 2020). This was consistent with results noted by Menezes et al. (2015), which suggested that the community assembly processes of fungi are more strongly influenced by the soil properties than that of bacteria.

Changes in soil properties under changing environments will directly affect plant performance and the associated microbial communities (Zhang et al., 2020). Soil salinity is a major factor that potentially alters the soil environment, and can also drive plant-soil feedbacks by modifying microbial communities (Bhat et al., 2020). This study showed that microbial indicator taxa underwent significant changes under salt-treated soil conditions. In the IS bacterial community, the dominance of Bacteroidetes and Actinobacteriota increased significantly (Supplementary Table 2). Moreover, RDA analysis confirmed that them to be highly correlated with soil salinity (Figure 6A), and their involvement in nutrient cycling because Actinobacteria form a potential source of bioactive secondary metabolites (Liu et al., 2014). In addition, *Massilia* not only served as a core taxon in IS, but also increased in abundance by 2.6-fold in IP (Supplementary Table 2). Yang et al. (2019) found that *Massilia* are capable of synthesizing a variety of secondary metabolites and enzymes, and exhibit greatly diverse functions, including phosphate solubilization, phenanthrene degradation, and heavy metal toxicity tolerance. This indicates that plants can recruit beneficial microbes under stress conditions, which in turn can enhance plants’ tolerance to adverse environmental stresses. Peanut-specific effects were observed on the fungal community profile. Entomophthoromycota and Kickxellomycota were specifically associated with peanut and highly susceptible to salinity. RDA analysis also showed that they were strongly correlated with the NH_4_^+^-N and AK contents (Figure 6B), which might be responsible for the low soil nutrient availability under salt stress. *Fusarium*, *Funneliformis*, and *Trechispora* were also sensitive to soil salinity, and their relative abundance increased significantly in SIP and SIS. *Fusarium* are significant pathogens that causes ear rot and stalk rotting in the field (Li et al., 2019). However, some researchers have shown *Funneliformis* to improve the salinity stress tolerance of *Arabidopsis thaliana* (Leguminosae) (Ren et al., 2016). Mukhtar et al. (2018) observed that plants can shape their rhizosphere bacterial communities and recruit environmental-stress-resistant bacteria. Therefore, when plants are subjected to pathogen infection under stress conditions, they can recruit beneficial microbes from the rhizosphere by self-regulation, in order to improve their stress tolerance. However, the specific mechanisms governing the assembly of the plant microbiome and its modulation according to plant growth environments are extremely complex and, to some extent, unpredictable.

Under salt-treated soil condition, the number of common OTUs among IP, II and IS were significantly reduced upon salt stress (Figures 2B,D), which implies that the richness and diversity of microbial communities were affected by changes in soil environmental factors. However, there were still species-specific differences in the community structure (Table 1), and the specific changes in the rhizosphere communities of different plant species have important implications in shaping microbial communities of II and improving ecosystem services of intercropping. Under salt-treated soil conditions, the mean relative abundance of Ascomycota in IP and II increased by 35.7%, whereas that of Glomeromycota in IP was significantly decreased, with it being significantly increased in IS and remaining essentially unchanged in II (Supplementary Table 3). Terrestrial plants and soil fungi of the phylum Glomeromycota have been reported to form arbuscular mycorrhizal (AM) symbiosis (Zhou et al., 2017), and the symbiotic structure has been reported to assist plants to absorb inorganic salts in the soil, which is critical for plants to adapt to the terrestrial environment (Kadowaki et al., 2018). These plants can also survive in high-salinity environments and form symbiotic relationships with epiphytes (Fellbaum et al., 2012). This illustrated the importance of interspecies complementation; sorghum with a strong salt tolerance could contribute to the overall maintenance of a microbial community structure by recruiting a balanced beneficial microbial community. At multiple taxonomic levels, the most predominant core taxa were observed at higher relative abundances in II than in IP or IS, with the abundance of *Talaromyces* being increased by 85.5% and that of *Sphingomonas* being increased by 65.5% (Supplementary Tables 2, 3). Studies have indicated that fungi, such as *Talaromyces*, are able to produce an abundant array of metabolites during their metabolic processes, which plays an important role in improving the environmental adaptability of crops. In addition, *Sphingomonas* forms the most abundant genus within the phylum Proteobacteria (Mukhtar et al., 2017), which can be utilized in the biodegradation of aromatic compounds, and have great application potential in the fields of environmental protection and biotechnology (Gou et al., 2008). This implies that II is a suitable habitat for beneficial microbes, and a large number of recruited beneficial microbes can exhibit a variety of ecological functions under stress conditions, which contribute to enhancing the plants’ tolerance in response to environmental stresses.

In addition to the alterations in the relative abundance or diversity of the core microbial community members, host phylogeny is also an important factor in shaping the microbial community structure (Wang et al., 2020). Under normal soil conditions, Planctomycetota represented the most abundant taxon in the IS. From the evolutionary perspective, Verrucomicrobiota and Planctomycetota showed more similar levels (Butler et al., 2007). Therefore, the high enrichment of Verrucomicrobiota in the II under salt stress may be the result of the evolution of Planctomycetota in IS. Functional RDA of environmental factors and the microbial community may also explain our results, which showed Planctomycetota to had a significant correlation with the soil AP content, whereas Verrucomicrobiota had a significant correlation with thevsoil TN content (Figure 6A and Supplementary Table 4). However, the functions of associations between nutrient availability and microorganisms are mostly unknown, and the biological implication behind microbial community succession and its influence on the intercropping system needs to be further elucidated.

## Conclusion

In conclusion, this study showed that soil salinity is a prerequisite for shaping soil microbial communities. Furthermore, peanut was a stronger driving force in shaping the microbial community of II than sorghum, and the bacterial phylogenetic community composition of IP was similar to that of II under different soil conditions. Although the main phyla of the fungal community in IP and IS were largely similar, the community differed in taxonomic composition. Under salt-treated soil conditions, interspecies interactions were beneficial to the host-specific recruitment of microorganisms, and hence contributed to driving the evolution of intrinsic microbial populations, ultimately leading to the specific microbial assemblage to combat different environmental stress conditions. However, this complex association mechanism and the crucial plant biological functions performed by beneficial microbes have not yet been clarified, and would needs to be elucidated by multidisciplinary research in the future. Moreover, whether these mechanisms could be applied to the natural ecosystems remains to be elucidated.

## Data Availability Statement

The datasets presented in this study can be found in online repositories. The names of the repository/repositories and accession number(s) can be found below: https://www.ncbi.nlm.nih.gov/bioproject/PRJNA707614; https://www.ncbi.nlm.nih.gov/bioproject/PRJNA707613.

## Author Contributions

XS designed the research, analyzed the data, and prepared the draft of the manuscript. XZ, CJ, and HZ contributed reagents and materials. JR, JD, QD, and CZ conducted the experiments. YZ reviewed drafts of the manuscript. HY organized and coordinated the whole project. All authors contributed to the intellectual input and assistance to this study and manuscript preparation.

## Conflict of Interest

The authors declare that the research was conducted in the absence of any commercial or financial relationships that could be construed as a potential conflict of interest.

## Footnotes

1 http://www.arb-silva.de

2 http://unite.ut.ee/index.php

3 http://www.mothur.org/

4 http://circos.ca/

5 http://huttenhower.sph.harvard.edu/lefse/

## Supplementary Material

Supplementary Figure 1 Planting patterns of peanut and sorghum. N, normal soil conditions; S, 0.25% salt-treated soil conditions; IP, peanut rhizosphere; II, interspecific interaction zone; IS, sorghum rhizosphere.

Supplementary Table 1 Microbial community sequencing data of the peanut rhizosphere (IP), sorghum rhizosphere (IS), and interspecific interaction zone (II) under different soil conditions (S, 0.25% salt-treated soil condition; N, normal soil condition).

Supplementary Table 2 Relative abundance of the peanut rhizosphere (IP), sorghum rhizosphere (IS), and interspecific interaction zone (II) bacterial communities under different soil conditions (S, 0.25% salt-treated soil condition; N, normal soil condition) at the phylum and genus levels.

Supplementary Table 3 Relative abundance of the peanut rhizosphere (IP), sorghum rhizosphere (IS), and interspecific interaction zone (II) fungal communities under different soil conditions (S, 0.25% salt-treated soil condition; N, normal soil condition) at the phylum and genus levels.

Supplementary Table 4 The nutrient contents of in the peanut rhizosphere (IP), sorghum rhizosphere (IS), and interspecific interaction zone (II) under different soil conditions (S, 0.25% salt-treated soil condition; N, normal soil condition).

## Abbreviations

AK: Available potassium
AN: Available nitrogen
AP: Available phosphorus
II: interspecific interaction zone
IP: peanut rhizosphere
IS: sorghum rhizosphere
ITS: internal transcribed spacer
LDA: Linear discriminant analysis
LEfSe: Linear discriminant analysis effect size
OTU: operational taxonomic units
PCoA: principal coordinate analysis
RDA: Redundancy analysis
TK: Total potassium
TN: Total nitrogen
TP: Total phosphorus
UPGMA: unweighted pair group method with arithmetic mean.
