# Supplementary material for: Influence of Peanut, Sorghum, and Soil Salinity on Microbial Community Composition in Interspecific Interaction Zone
Source: Front Microbiol. 2021 May 24;12:678250. doi: 10.3389/fmicb.2021.678250 (PMC8180576; doi:10.3389/fmicb.2021.678250)
Supplement: Supplementary Figure 1 — Planting patterns of peanut and sorghum. N, normal soil conditions; S, 0.25% salt-treated soil conditions; IP, peanut rhizosphere; II, interspecific interaction zone; IS, sorghum rhizosphere. [file Data_Sheet_1.docx]

***Supplementary Material***

**Supplementary Figures**


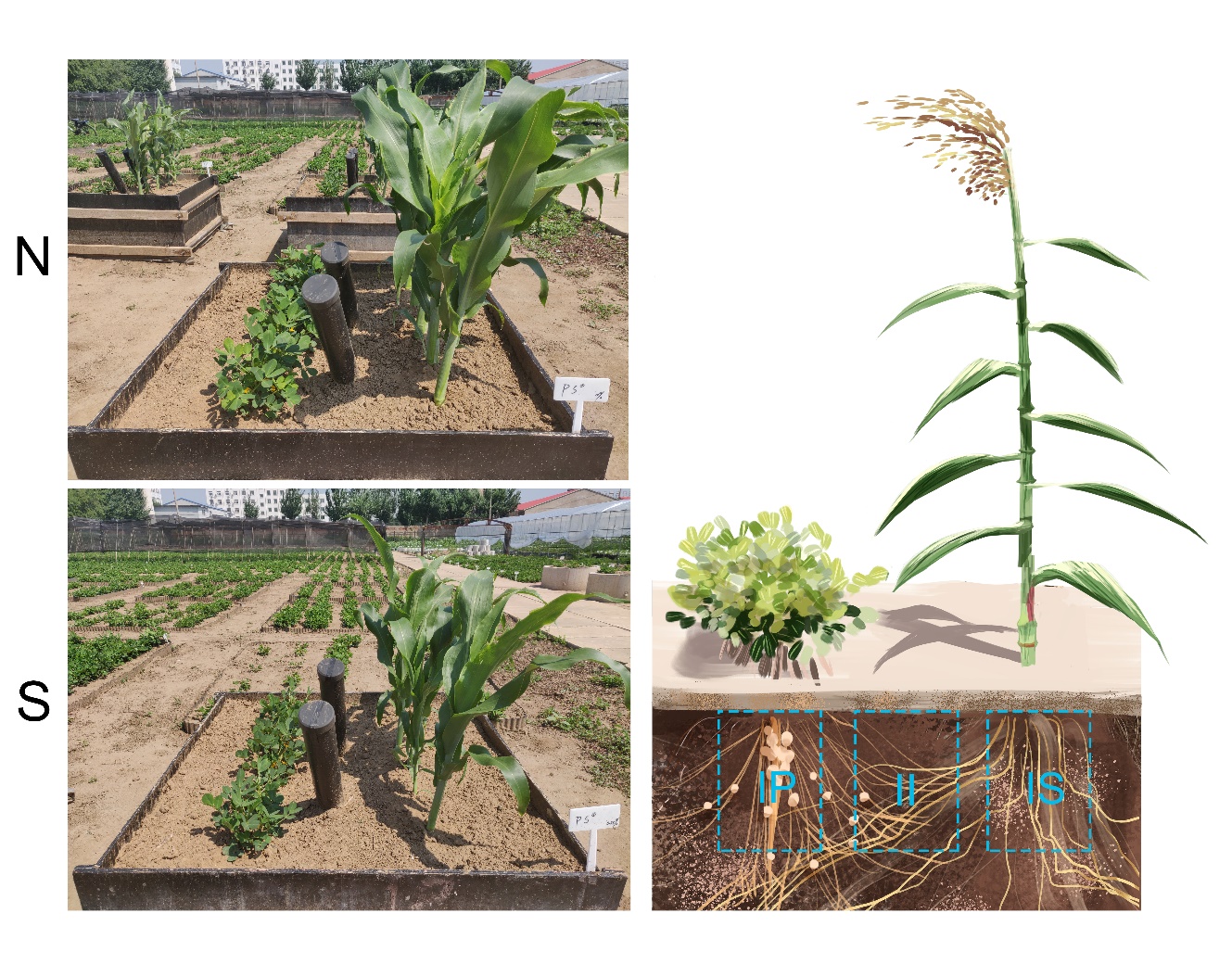


**Figure S1.** Planting patterns of peanut and sorghum. N, normal soil conditions; S, 0.25% salt-treated soil conditions; IP, peanut rhizosphere; II, interspecific interaction zone; IS, sorghum rhizosphere.

**Supplementary Tables**

**Table S1** Microbial community sequencing data of the peanut rhizosphere (IP), sorghum rhizosphere (IS), and interspecific interaction zone (II) under different soil conditions (S, 0.25% salt-treated soil condition; N, normal soil condition).

| **Microorganism** | **Sample** | **Barcode-CCS** | **Filtered-CCS** | **Optimization-CCS** | **AvgLen(bp)** | **Effective (%)** |
| --- | --- | --- | --- | --- | --- | --- |
| **Bacteria** | SII1 | 20888 | 20797 | 20034 | 1455 | 95.91 |
|  | SII2 | 21095 | 21000 | 20233 | 1455 | 95.91 |
|  | SII3 | 21091 | 21014 | 20311 | 1455 | 96.3 |
|  | SIP1 | 20945 | 20871 | 19787 | 1454 | 94.47 |
|  | SIP2 | 21200 | 21162 | 20324 | 1453 | 95.87 |
|  | SIP3 | 20970 | 20853 | 19656 | 1453 | 93.73 |
|  | SIS1 | 21010 | 20860 | 19654 | 1455 | 93.55 |
|  | SIS2 | 20709 | 20599 | 19523 | 1455 | 94.27 |
|  | SIS3 | 21148 | 21034 | 19834 | 1455 | 93.79 |
|  | NII1 | 19854 | 19773 | 19133 | 1457 | 96.37 |
|  | NII2 | 21005 | 20929 | 20293 | 1457 | 96.61 |
|  | NII3 | 21014 | 20921 | 20338 | 1457 | 96.78 |
|  | NIP1 | 20985 | 20876 | 20137 | 1458 | 95.96 |
|  | NIP2 | 20889 | 20790 | 20115 | 1456 | 96.29 |
|  | NIP3 | 21028 | 20935 | 20223 | 1456 | 96.17 |
|  | NIS1 | 20623 | 20508 | 19477 | 1456 | 94.44 |
|  | NIS2 | 20611 | 20527 | 19381 | 1455 | 94.03 |
|  | NIS3 | 20975 | 20848 | 19818 | 1455 | 94.48 |
| **Fungi** | SII1 | 8067 | 8035 | 8018 | 618 | 99.39 |
|  | SII2 | 8007 | 7934 | 7920 | 629 | 98.91 |
|  | SII3 | 7936 | 7873 | 7850 | 636 | 98.92 |
|  | SIP1 | 7975 | 7912 | 7717 | 611 | 96.76 |
|  | SIP2 | 8051 | 7984 | 7949 | 625 | 98.73 |
|  | SIP3 | 8039 | 7741 | 7721 | 630 | 96.04 |
|  | SIS1 | 7929 | 7869 | 7520 | 611 | 94.84 |
|  | SIS2 | 8005 | 7966 | 7872 | 610 | 98.34 |
|  | SIS3 | 8031 | 7925 | 7718 | 609 | 96.1 |
|  | NII1 | 7973 | 7802 | 7780 | 638 | 97.58 |
|  | NII2 | 7949 | 7898 | 7894 | 631 | 99.31 |
|  | NII3 | 8030 | 7756 | 7737 | 635 | 96.35 |
|  | NIP1 | 8109 | 8081 | 8046 | 641 | 99.22 |
|  | NIP2 | 7937 | 7874 | 7853 | 622 | 98.94 |
|  | NIP3 | 8045 | 7952 | 7937 | 629 | 98.66 |
|  | NIS1 | 8015 | 7959 | 7946 | 637 | 99.14 |
|  | NIS2 | 8031 | 7948 | 7926 | 640 | 98.69 |
|  | NIS3 | 7911 | 7830 | 7791 | 615 | 98.48 |

**Table S2** Relative abundance of the peanut rhizosphere (IP), sorghum rhizosphere (IS), and interspecific interaction zone (II) bacterial communities under different soil conditions (S, 0.25% salt-treated soil condition; N, normal soil condition) at the phylum and genus levels.

| **Taxonomy** | **Bacteria** | **SII** | **SIP** | **SIS** | **NII** | **NIP** | **NIS** |
| --- | --- | --- | --- | --- | --- | --- | --- |
| **Phylum** | Proteobacteria | 0.3327 | 0.3675 | 0.3812 | 0.3272 | 0.3866 | 0.3900 |
|  | Bacteroidota | 0.0861 | 0.1211 | 0.1261 | 0.0778 | 0.0807 | 0.1112 |
|  | Acidobacteriota | 0.0982 | 0.0836 | 0.0736 | 0.1105 | 0.1012 | 0.0824 |
|  | Verrucomicrobiota | 0.0878 | 0.0752 | 0.0722 | 0.0712 | 0.0551 | 0.0635 |
|  | Actinobacteriota | 0.0610 | 0.0655 | 0.0706 | 0.0475 | 0.0524 | 0.0513 |
|  | Gemmatimonadota | 0.0597 | 0.0484 | 0.0441 | 0.0584 | 0.0517 | 0.0520 |
|  | Planctomycetota | 0.0486 | 0.0501 | 0.0607 | 0.0475 | 0.0425 | 0.0614 |
|  | Acidobacteria | 0.0341 | 0.0277 | 0.0264 | 0.0394 | 0.0326 | 0.0247 |
|  | Myxococcota | 0.0254 | 0.0228 | 0.0199 | 0.0332 | 0.0280 | 0.0255 |
|  | Nitrospirota | 0.0180 | 0.0133 | 0.0107 | 0.0214 | 0.0208 | 0.0159 |
|  | Others | 0.0431 | 0.0497 | 0.0506 | 0.0391 | 0.0349 | 0.0468 |
|  | Unclassified | 0.1053 | 0.0752 | 0.0642 | 0.1268 | 0.1133 | 0.0753 |
| **Genus** | Vicinamibacter | 0.0450 | 0.0376 | 0.0386 | 0.0561 | 0.052 | 0.0392 |
|  | Sphingomonas | 0.0581 | 0.0468 | 0.0445 | 0.0351 | 0.0334 | 0.0318 |
|  | Gemmatimonas | 0.0453 | 0.036 | 0.0321 | 0.0455 | 0.0421 | 0.0392 |
|  | Massilia | 0.0087 | 0.0332 | 0.0670 | 0.0060 | 0.0092 | 0.0655 |
|  | Candidatus_Udaeobacter | 0.0451 | 0.028 | 0.0243 | 0.0375 | 0.0225 | 0.0207 |
|  | Brevitalea | 0.0290 | 0.0235 | 0.0224 | 0.0325 | 0.0258 | 0.0212 |
|  | Flavisolibacter | 0.0201 | 0.0278 | 0.0354 | 0.0141 | 0.0151 | 0.0285 |
|  | Tepidisphaera | 0.0225 | 0.0248 | 0.0283 | 0.0188 | 0.0156 | 0.0278 |
|  | Pedosphaera | 0.0214 | 0.0217 | 0.0213 | 0.0174 | 0.0151 | 0.0164 |
|  | Arenimonas | 0.0132 | 0.0178 | 0.0233 | 0.0163 | 0.0216 | 0.0208 |
|  | Others | 0.3581 | 0.4382 | 0.4136 | 0.3451 | 0.4001 | 0.4218 |
|  | Unclassified | 0.3334 | 0.2647 | 0.2492 | 0.3757 | 0.3475 | 0.2670 |

**Table S3** Relative abundance of the peanut rhizosphere (IP), sorghum rhizosphere (IS), and interspecific interaction zone (II) fungal communities under different soil conditions (S, 0.25% salt-treated soil condition; N, normal soil condition) at the phylum and genus levels.

| **Taxonomy** | **Fungi** | **SII** | **SIP** | **SIS** | **NII** | **NIP** | **NIS** |
| --- | --- | --- | --- | --- | --- | --- | --- |
| **Phylum** | Ascomycota | 0.4242 | 0.4483 | 0.4874 | 0.3165 | 0.3264 | 0.4339 |
|  | Basidiomycota | 0.1684 | 0.3120 | 0.2218 | 0.2132 | 0.1836 | 0.2595 |
|  | Glomeromycota | 0.1358 | 0.0772 | 0.1366 | 0.1372 | 0.1466 | 0.0677 |
|  | Mucoromycota | 0.0835 | 0.0756 | 0.0631 | 0.1124 | 0.1077 | 0.0985 |
|  | Chytridiomycota | 0.0959 | 0.0123 | 0.0201 | 0.0520 | 0.0609 | 0.0510 |
|  | Mortierellomycota | 0.0443 | 0.0216 | 0.0326 | 0.0994 | 0.0549 | 0.0389 |
|  | Entomophthoromycota | 0.0000 | 0.0000 | 0.0000 | 0.0015 | 0.0069 | 0.0000 |
|  | Zoopagomycota | 0.0007 | 0.0002 | 0.0003 | 0.0017 | 0.0013 | 0.0008 |
|  | Kickxellomycota | 0.0002 | 0.0000 | 0.0004 | 0.0016 | 0.0014 | 0.0010 |
|  | Cryptomycota | 0.0000 | 0.0003 | 0.0002 | 0.0005 | 0.0003 | 0.0001 |
|  | Others | 0.0000 | 0.0000 | 0.0000 | 0.0000 | 0.0000 | 0.0000 |
|  | Unclassified | 0.0471 | 0.0527 | 0.0375 | 0.0639 | 0.1100 | 0.0486 |
| **Genus** | Talaromyces | 0.2308 | 0.2066 | 0.1984 | 0.0328 | 0.0386 | 0.1366 |
|  | Mortierella | 0.1083 | 0.0447 | 0.0679 | 0.1933 | 0.1420 | 0.1329 |
|  | Fusarium | 0.0199 | 0.0493 | 0.0633 | 0.0243 | 0.0130 | 0.0488 |
|  | Funneliformis | 0.0182 | 0.0524 | 0.0278 | 0.0175 | 0.0184 | 0.0045 |
|  | Glomus | 0.0202 | 0.0067 | 0.0335 | 0.040 | 0.0206 | 0.0121 |
|  | Trechispora | 0.0140 | 0.049 | 0.0212 | 0.0212 | 0.0185 | 0.0062 |
|  | Rhizophagus | 0.0241 | 0.0215 | 0.0258 | 0.0173 | 0.0223 | 0.0097 |
|  | Septoglomus | 0.0177 | 0.0098 | 0.0153 | 0.0318 | 0.0174 | 0.0207 |
|  | Alternaria | 0.0076 | 0.020 | 0.0264 | 0.0036 | 0.0391 | 0.0149 |
|  | Conocybe | 0.0026 | 0.005 | 0.0159 | 0.0032 | 0.0238 | 0.0594 |
|  | Others | 0.2626 | 0.3734 | 0.3368 | 0.3636 | 0.3669 | 0.3429 |
|  | Unclassified | 0.2741 | 0.1618 | 0.1679 | 0.2514 | 0.2794 | 0.2113 |

**Table S4** The nutrient contents of in the peanut rhizosphere (IP), sorghum rhizosphere (IS), and interspecific interaction zone (II) under different soil conditions (S, 0.25% salt-treated soil condition; N, normal soil condition).

|  | **TN** | **TP** | **TK** | **NH_4_^+^-N** | **AP** | **AK** | **C/N ratio** |
| --- | --- | --- | --- | --- | --- | --- | --- |
| **SIP** | 3406.11±82.35^a^ | 402.22±29.12^b^ | 5739.50±282.26^a^ | 55.01±1.10^b^ | 25.98±0.29^a^ | 321.15±2.45^c^ | 10.4±0.23^bc^ |
| **SII** | 3723.33±834.38^a^ | 381.11±25.89^b^ | 5280.36±276.73^ab^ | 55.39±1.81^b^ | 20.37±0.95^c^ | 350.00±1.50^b^ | 10.18±0.29^c^ |
| **SIS** | 3301.11±95.60^a^ | 458.89±47.30^ab^ | 5882.98±203.42^a^ | 54.66±0.88^b^ | 20.49±1.54^c^ | 326.80±17.27^bc^ | 10.49±0.03^ab^ |
| **NIP** | 3342.78±21.17^a^ | 446.67±42.56^ab^ | 4821.22±179.21^bc^ | 59.58±0.53^a^ | 21.32±0.58^bc^ | 384.53±7.99^a^ | 10.77±0.02^a^ |
| **NII** | 3254.44±100.83^a^ | 511.11±84.74^a^ | 4620.35±507.48^bc^ | 58.95±1.43^a^ | 21.61±0.29^bc^ | 377.23±10.51^a^ | 10.13±0.18^c^ |
| **NIS** | 3280.00±38.33^a^ | 405.56±30.25^b^ | 4419.48±388.19^c^ | 59.18±4.17^a^ | 22.36±0.96^b^ | 381.63±24.54^a^ | 10.65±0.20^ab^ |

*Different lowercase letters in the same column indicate significant differences between the groups at a significance level of p < 0.05.*
